# Brain mGlu5 Is Linked to Cognition and Cigarette Smoking but Does Not Differ From Control in Early Abstinence From Chronic Methamphetamine Use

**DOI:** 10.1093/ijnp/pyae031

**Published:** 2024-08-09

**Authors:** Megan N McClintick, Robert M Kessler, Mark A Mandelkern, Tarannom Mahmoudie, Daicia C Allen, Hilary Lachoff, Jean-Baptiste F Pochon, Dara G Ghahremani, Judah B Farahi, Edwin Partiai, Robert A Casillas, Larissa J Mooney, Andy C Dean, Edythe D London

**Affiliations:** Veterans Administration of Greater Los Angeles System, Los Angeles, California, USA; Semel Institute and Department of Psychiatry and Biobehavioral Sciences, University of California Los Angeles, Los Angeles, California, USA; Semel Institute and Department of Psychiatry and Biobehavioral Sciences, University of California Los Angeles, Los Angeles, California, USA; Veterans Administration of Greater Los Angeles System, Los Angeles, California, USA; Department of Physics, University of California Irvine, Irvine, California, USA; Semel Institute and Department of Psychiatry and Biobehavioral Sciences, University of California Los Angeles, Los Angeles, California, USA; Veterans Administration of Greater Los Angeles System, Los Angeles, California, USA; Semel Institute and Department of Psychiatry and Biobehavioral Sciences, University of California Los Angeles, Los Angeles, California, USA; Semel Institute and Department of Psychiatry and Biobehavioral Sciences, University of California Los Angeles, Los Angeles, California, USA; Semel Institute and Department of Psychiatry and Biobehavioral Sciences, University of California Los Angeles, Los Angeles, California, USA; Veterans Administration of Greater Los Angeles System, Los Angeles, California, USA; Veterans Administration of Greater Los Angeles System, Los Angeles, California, USA; Veterans Administration of Greater Los Angeles System, Los Angeles, California, USA; Veterans Administration of Greater Los Angeles System, Los Angeles, California, USA; Semel Institute and Department of Psychiatry and Biobehavioral Sciences, University of California Los Angeles, Los Angeles, California, USA; Semel Institute and Department of Psychiatry and Biobehavioral Sciences, University of California Los Angeles, Los Angeles, California, USA; Veterans Administration of Greater Los Angeles System, Los Angeles, California, USA; Semel Institute and Department of Psychiatry and Biobehavioral Sciences, University of California Los Angeles, Los Angeles, California, USA

**Keywords:** Group-I metabotropic glutamate receptor subtype 5, stimulant use disorder, dorsolateral prefrontal cortex, positron emission tomography, verbal learning

## Abstract

**Background:**

The group-I metabotropic glutamate receptor subtype 5 (mGlu5) has been implicated in methamphetamine exposure in animals and in human cognition. Because people with methamphetamine use disorder (MUD) exhibit cognitive deficits, we evaluated mGlu5 in people with MUD and controls and tested its association with cognitive performance.

**Methods:**

Positron emission tomography was performed to measure the total V_T_ of [^18^F]FPEB, a radiotracer for mGlu5, in brains of participants with MUD (abstinent from methamphetamine for at least 2 weeks, N = 14) and a control group (N = 14). Drug use history questionnaires and tests of verbal learning, spatial working memory, and executive function were administered. Associations of V_T_ with methamphetamine use, tobacco use, and cognitive performance were tested.

**Results:**

MUD participants did not differ from controls in global or regional V_T_, and measures of methamphetamine use were not correlated with V_T_. V_T_ was significantly higher globally in nonsmoking vs smoking participants (main effect, *P* = .0041). MUD participants showed nonsignificant weakness on the Rey Auditory Verbal Learning Task and the Stroop test vs controls (*P *=* *.08 and *P* = .13, respectively) with moderate to large effect sizes, and significantly underperformed controls on the Spatial Capacity Delayed Response Test (*P *=* *.015). Across groups, Rey Auditory Verbal Learning Task performance correlated with V_T_ in the dorsolateral prefrontal cortex and superior frontal gyrus.

**Conclusion:**

Abstinent MUD patients show no evidence of mGlu5 downregulation in brain, but association of V_T_ in dorsolateral prefrontal cortex with verbal learning suggests that medications that target mGlu5 may improve cognitive performance.

**Significance Statement**

We examined mGlu5 in the brains of participants with MUD and controls using PET with [^18^F]FPEB, a selective radiotracer for mGlu5. Although the total volume of distribution (V_T_) of [^18^F]FPEB in brain showed no significant group difference and no relation to indices of methamphetamine use, cigarette smoking was associated with lower V_T_ globally across all participants. V_T_ in the prefrontal cortex correlated with verbal learning, suggesting that mGlu5 may be a useful therapeutic target to improve cognition in MUD.

## INTRODUCTION

Methamphetamine use disorder (MUD) is a major public health concern as part of the current overdose and broader public health crisis involving illicit drug use (Rawson et al., 2023). From 1999 to 2021, the methamphetamine mortality rate increased 50-fold among US residents aged 15 to 74 years (Hoopsick and Yockey, 2023). People who use methamphetamine chronically exhibit deficits in several cognitive domains (e.g., Dean et al., 2013; Potvin et al., 2018), including attention; verbal learning; inhibitory control; reward processing; social cognition; and verbal, visual, and working memory. As there is no FDA-approved medication for the treatment of MUD, behavioral approaches are the mainstay of therapy. However, the presence of cognitive impairments can thwart engagement in behavioral treatments and predict poor outcome (Brorson et al., 2013).

While monoamines such as dopamine play a key role in the initiation of and psychomotor effects of psychostimulant use, metabotropic glutamate receptors influence the regulation of dopamine release in these processes (Arai et al., 1996, 1997; Shimazoe et al., 2002). The group-I metabotropic glutamate receptor subtype 5 (mGlu5) is a predominantly postsynaptic receptor that contributes to excitatory neurotransmission (Shigemoto et al., 1993, 1997; Daggett et al., 1995; Gereau and Conn, 1995). Within the brain, its expression is highest in the hippocampus, cerebral cortex, striatum, and thalamus and lowest in the cerebellum (Daggett et al., 1995). Repeated exposure to methamphetamine increases mGlu5 receptor signaling (Szumlinski et al., 2017) and levels in the nucleus accumbens in mice (Wright et al., 2016), and mice selectively bred for high methamphetamine intake have higher methamphetamine-evoked increases in extracellular glutamate, higher levels of mGlu5 in the nucleus accumbens, and higher basal extracellular glutamate levels in the medial prefrontal cortex than mice bred for low intake (Lominac et al., 2016; Szumlinski et al., 2017).

The mGlu5 is implicated in cognition (Homayoun and Moghaddam, 2010; Hullinger et al., 2015), particularly in memory processes (Lu et al., 1997; Bortolotto et al., 2005; Lee et al., 2005; Manahan-Vaughan and Braunewell, 2005; Bikbaev et al., 2008; Neyman and Manahan-Vaughan, 2008). Consistent with evidence of the procognitive effects of positive allosteric modulators of mGlu5 preclinically (Liu et al., 2008; Reichel et al., 2011), several recent studies utilizing positron emission tomography (PET) found positive associations between cognitive performance and mGlu5 availability in brain. Across participants with Alzheimer disease and healthy controls, global cognition scores were positively associated with hippocampal mGlu5 availability measured with [^18^F]PSS232 (Wang et al., 2024) and [^18^F]FPEB (Mecca et al., 2020). Higher mGlu5 binding potential in the caudate and left temporal cortex, measured with [^11^C]ABP688, correlated with faster processing speed in individuals with schizophrenia (Régio Brambilla et al., 2020).

The mGlu5 has not been assessed in people with MUD, but studies using PET with [^11^C]ABP688 have indicated that cocaine-dependent participants have below-control mGlu5 availability throughout the brain (Martinez et al., 2014; Milella et al., 2014). Here we used PET with [^18^F]FPEB, a radioligand with high selectivity and specificity for the negative allosteric modulator site on mGlu5 (Wong et al., 2013; Park et al., 2015), to measure the total volume of distribution (V_T_) of [^18^F]FPEB in brains of individuals with MUD and matched control participants. In light of the global deficit in mGlu5 observed in cocaine-dependent participants (Martinez et al., 2014; Milella et al., 2014), we hypothesized that V_T_ for [^18^F]FPEB would be lower in MUD compared with controls. Given prior literature showing positive relationships between cognitive function and mGlu5 binding and the importance of the prefrontal cortex in higher-level cognitive functions, we also expected V_T_ in the prefrontal cortex to be related to cognitive performance across participants. Lastly, prior studies have shown lower mGlu5 availability in the brains of people who smoked cigarettes compared with values in nonsmokers (Akkus et al., 2013, 2017; Hulka et al., 2014; Baldassarri et al., 2023). We therefore tested the interaction between smoking and MUD on V_T_ for [^18^F]FPEB.

## MATERIALS AND METHODS

### Participants

Fourteen individuals who met DSM-5 criteria for severe MUD (4 women and 10 men) and 14 control participants (5 women and 9 men) underwent PET scans with [^18^F]FPEB to measure mGlu5 V_T_ in brain. MUD group participants were recruited from residential (N = 12) and outpatient treatment programs (N = 2) and were abstinent from methamphetamine for at least 14 but fewer than 75 days (mean = 42 days). Control participants were recruited from the community via online and print advertisements. All procedures were approved by the University of California Los Angeles Institutional Review Board and the Greater Los Angeles Veterans Affairs Institutional Review Board and Radiation Safety Committee. All participants were fluent in English and signed informed consent documents after receiving a detailed description of the protocol. Exclusion criteria, determined by a psychiatric evaluation using the Mini International Neuropsychiatric Interview for DSM-5 and a physician-conducted history, physical examination and laboratory tests, included: neurological, cardiovascular, pulmonary, endocrine, autoimmune, untreated renal hepatic, or active infectious disease; psychotic disorders; suicidality; asthma; age outside the range of 18 to 65 years inclusive; MRI contraindications (metallic implants, claustrophobia); pregnancy; head trauma with loss of consciousness >30 minutes; current use of prescribed psychotropic medications with dopaminergic or glutamatergic action; or meeting prisoner status per California Section 3502. Further exclusions for the control group were the use of any psychotropic medications, meeting criteria for a substance use disorder, or more than 5 lifetime uses of substances of abuse other than nicotine, caffeine, or cannabis. Several MUD group participants met criteria for additional substance use disorders: severe opioid use disorder (N = 1), severe alcohol use disorder (N = 4), and severe sedative use disorder (N = 1). Some also met criteria for additional diagnoses, including past depressive episodes (N = 4), current anxiety disorders (N = 2), bulimia nervosa (N = 2), obsessive compulsive disorder (N = 1), panic disorder (N = 1), post-traumatic stress disorder (PTSD; N = 1), and antisocial personality disorder (N = 8). In the control group, some participants met criteria for past depressive episodes (N = 6), anxiety disorder (N = 1), and antisocial personality disorder (N = 2). MUD group participants reported significantly fewer years of education than the control group (Table 1).

**Table 1.** Participant Characteristics (Mean ± SE)

	Control		Methamphetamine use disorder^*a*^	
	Smoking N = 6	Nonsmoking N = 8	Smoking N = 8	Nonsmoking N = 6
Sex (men/women)	5/1	4/4	5/3	5/1
Age (y)	32.0 ± 3.6	33.0 ± 2.8	36.3 ± 2.0	33.5 ± 3.3
Parent’s education (y)^*b*^	15.7 ± 1.1	13.8 ± 2.0	12.5 ± 0.6	14.0 ± 2.0
Education (y)^*c*^^*,*^^*d*^	15.8 ± 0.8	15.9 ± 0.7	11.7 ± 0.5	13.8 ± 0.7
FSIQ	107 ± 3.0	106 ± 3.4	101 ± 3.4	103 ± 2.1
Race (ethnicity)				
Caucasian (non-Hispanic)	4	1	1	2
Caucasian (Hispanic)	0	3	3	2
African American	1	2	1	0
Asian	0	1	0	0
Hawaiian/Pacific Islander	1	0	0	0
American Indian/Alaska Native	0	0	0	0
More than 1 race	0	1	3	2
Handedness (right-handed)	6	8	6	6

Abbreviations: FSIQ, full scale intelligence quotient; MUD, methamphetamine use disorder.

^*a*^Participants met DSM-5 criteria for MUD.

^*b*^Missing data: 1 control, 6 MUD.

^*c*^Missing data: 1 control, 1 MUD.

^*d*^ *P *=* *.0002.

Although there were no other significant group differences in demographic and drug use variables, the MUD group had more participants who smoked cigarettes than the control group. Among those who smoked, nicotine dependence (Fagerström test scores) and withdrawal on the test day (Shiffman-Jarvik Withdrawal scores) were modest (Table 2). To standardize the timing of nicotine and cannabis exposure across all participants and prevent the effect of nicotine on mGlu5, all participants were instructed to abstain from using tobacco products for at least 12 hours and from cannabis products for at least 48 hours before PET and MRI scans. The average duration of abstinence from tobacco at the time of scanning was nonsignificantly higher in the control group because 1 participant had abstained for 17 days; all other participants in both groups had abstained between 16 and 25 hours before PET. Abstinence from smoking was confirmed by carbon monoxide measurements of <10 ppm or a 50% reduction from baseline intake, determined with a smokerlyzer (Covita Micro+ basic). Nicotine replacement therapies were not used by any participants.

**Table 2.** Self-reported Substance Use (Mean ± SE)^*a*^

	Control		Methamphetamine use disorder	
	Smoking N = 6	Non-smoking N = 8	Smoking N = 8	Non-smoking N = 6
Methamphetamine use				
Duration (y)			6.52 ± 2.45	8.28 ± 3.13
Average daily use (g/d)			2.6 ± 1.7	1.3 ± 0.4
Days used in last 30 d			21.6 ± 4.6	9.7 ± 3.2
Duration of abstinence (d)			38.9 ± 6.0	45.2 ± 5.8
Route of administration				
Smoking, n (%)			7 (87.5)	5 (83.3)
Intravenous injection, n (%)			0 (0)	2 (33.3)
Sniff/snort, n (%)			3 (37.5)	5 (83.3)
Tobacco use				
Days used in last 30 d	17.5 ± 4.3		26.3 ± 2.1	
Cigarettes per day	7.3 ± 2.9		7.1 ± 2.0	
Duration of abstinence (h)	1063.6 ± 40.7		18.8 ± 1.0	
Nicotine withdrawal (Shiffman- Jarvik average score)	3.3 ± 0.7		3.2 ± 0.5	
Fagerström (FTND) score	2.8 ± 1.4		2.4 ± 0.8	
Cannabis use				
Number of participants, n (%)	2 (33.3)	1 (12.5)	3 (37.5)	2 (33.3)
Days used in last 30 d	12 ± 8	5	13.0 ± 8.6	1.5 ± 0.5
Alcohol use				
Number of participants, n (%)	6 (100)	4 (50)	3 (37.5)	2 (33.3)
Days used in last 30 d	7.7 ± 2.2	4.2 ± 2.1	10.3 ± 6.6	9.5 ± 5.5

Abbreviations: FTND, Fagerström test for nicotine dependence.

^*a*^No significant differences in tobacco, cannabis, or alcohol use.

MUD group participants were required to maintain abstinence from other substances for at least 4 days before PET and MRI scans, demonstrated by negative urine toxicology (methamphetamine, amphetamine, opiates, cocaine, and benzodiazepines) and breathalyzer (alcohol) tests. Control participants were required to test negative for all substances except cannabis and nicotine. A single participant in the MUD tested positive for cannabis use on scanning and cognitive testing days and had been abstinent from using cannabis products for more than 2 weeks at the time of testing. Tobacco use was not restricted on nonscanning study days, including cognitive testing days.

### Behavioral Assessments

All participants completed several interviews and self-report questionnaires to characterize their substance use and health history. These included a neurological history questionnaire used to ascertain study eligibility, and a 147-item assessment of current and past use of addictive substances. In addition, a 26-item questionnaire queried current and past methamphetamine use, and a 10-item questionnaire, adapted from the Cocaine Craving Questionnaire–Brief (Sussner et al., 2006), was used to assess current craving for methamphetamine. Participants who indicated that they smoked cigarettes also were administered the Fagerström Test for Nicotine Dependence (Heatherton et al., 1991) during screening and the Shiffman-Jarvik Withdrawal Scale (Shiffman and Jarvik, 1976) immediately before PET scanning.

We administered cognitive tests on a nonscanning day. These included the Rey Auditory Verbal Learning Test (RAVLT; Rey, 1941), which evaluates verbal retention, encoding, and retrieval ability. The variable of interest was immediate memory (age-adjusted Z-score trials A1-A5 total score). The Spatial Capacity Delayed Response Test (SCAP; based on Sternberg 1969; Glahn et al., 2003) was administered to test visual working memory. The primary outcome variable for this test was overall response accuracy, calculated through signal detection procedures (i.e., d-prime). Finally, the Stroop test (Delis et al., 2001) was administered as a test of inhibitory control. The primary outcome variable computed from the 3 subsections was the interference score, calculated as Stroop Color-Word Inhibition test score—(Stroop Color-Naming test score * Stroop Word-Reading test score)/Stroop Color-Naming test score + Stroop Word-Reading test score). The Wechsler test for Adult Reading (Wechsler, 2001) was used to produce an estimated Full-Scale Intelligence Quotient. Participants were allowed to smoke throughout the day as needed.

### Image Acquisition and Data Analyses

#### PET Acquisition

[^18^F]FPEB was produced via no-carrier-added [^18^F]fluorination of 3-nitro-5-[2-(pyridinyl)ethynyl]benzonitrile, a precursor for [^18^F]FPEB, followed by solid phase extraction purification and high-performance liquid chromatography (HPLC) purification (Lim et al., 2014). [^18^F]FPEB was i.v. administered as a bolus plus continuous infusion with *K*_bol_ = 205 minutes to achieve a steady state (Kessler et al., 2014a). The radiotracer dose was 5 mCi ± 10% error (mean = 5.09 ± 0.09) mCi (Supplemental Table 2). Continuous infusion over 140 minutes was ensured via the use of an infusion pump (Graseby 3400 Syringe Pump). In a past study in humans, a *K*_bol_ of 205 minutes produced good stability of total uptake at 90 to 130 minutes in high-uptake regions, including the hippocampus (Kessler et al., 2014b).

PET-imaging data were acquired with a Siemens Biograph mCT PET/CT scanner (Hoffman Estates, IL). Dynamic scanning for the acquisition of emission data began 110 minutes after [^18^F]FPEB bolus administration, lasting for a total of 30 minutes and consisting of three 10-minute frames. The data were acquired within a 2-hour period in the afternoon for all participants to minimize effects of within-day variation on mGlu5 (DeLorenzo et al., 2017). A computed tomography (CT) transmission scan was acquired immediately before dynamic scanning for attenuation correction. Four 8-mL venous samples were collected at 10-minute intervals during the equilibrium period (109, 119, 129, and 139 minutes after the start of [^18^F]FPEB administration). To measure total plasma radioactivity in the venous samples at equilibrium, the heparinized samples were centrifuged for 10 minutes at 3,000 × g, and activities in 0.2-mL plasma aliquots were determined. Activity was decay-corrected to the start of PET dynamic scanning and was corrected for the fraction of plasma radioactivity represented by unmetabolized parent. To calculate the percent parent [^18^F]FPEB, 2-mL plasma aliquots from each venous sample were mixed with acetonitrile. The resulting supernatant was analyzed by high-performance liquid chromatography via a Luna 5u C18(2) 100A 250 × 10 mm 5 micron Phenomenex column with a 2-mL loop and a mobile phase of 70/30 methanol/water with 0.1% triethylamine at a flow rate of 4 mL/min. Serial samples were collected every 30 seconds for 12 minutes and the activity counted. The unmetabolized parent fraction was determined as the ratio of the sum of activity in fractions containing the parent compound to the total amount of activity collected in all samples.

#### MRI Acquisition

Magnetic resonance image (MRI) data were acquired to guide anatomical sampling of the PET data. MR data were acquired with a 3-Tesla PRISMA MRI scanner with a weighted gradient-echo (MPRAGE) sequence, including the following parameters: TE = 2.24 ms, TI = 1060 ms, TR = 2400 ms, FOV = 240 256 mm^2^, flip angle = 8°, 745 Hz/pixel bandwidth, voxel size = 0.8 mm^3^, 208 sagittal slices.

#### PET Image Processing and Quantification

The PET acquisition comprised 3 contiguous frames, each consisting of 10 one-minute scans. The data were reconstructed using TrueX with Time of Flight (5 iterations, 21 subsets) to 30 one-minute volumes (400 × 400 × 109). Measured attenuation correction, relative scatter correction, and 2.0 mm Gaussian smoothing were applied.

Within each frame, the 10 volumes were realigned to the averaged volume for that frame using FSL FLIRT (Jenkinson et al., 2012) with a normalized correlation cost function. The averaged volumes were recomputed, and those for frames 2 and 3 were realigned to that for frame 1. The realigned averaged volumes for all 3 frames underwent co-registration and resampling to the structural MPRAGE image using FSL FLIRT with mutual information as the cost function. Visual inspection confirmed proper alignment and co-registration.

The structural MPRAGE image underwent segmentation via Freesurfer (Fischl et al., 2002; version 6.0.0) and was normalized to MNI space using linear (FSL FLIRT) and nonlinear (FSL FNIRT) registration. Volumes of interest (VOIs) from the FreeSurfer-based Desikan-Killiany Atlas in MNI space transformed to native space. For voxelwise analyses, the aligned co-registered averaged PET volumes were warped from native space to MNI space.

The primary outcome measure of the [^18^F]FPEB data was the total V_T_, computed as the ratio of [^18^F]FPEB activity in tissue (brain) to venous plasma parent activity (Innis et al., 2007). Specifically, V_T_ was calculated as the ratio of activity in brain tissue in each 10-minute frame to the mean activity in venous samples measured immediately before and after the frame. V_T_ was averaged within each VOI in native space. Bilateral VOIs were used for all analyses. The dorsolateral prefrontal cortex VOI was comprised of the rostral middle frontal and caudal middle frontal VOIs. The inferior frontal gyrus VOI was comprised of the pars opercularis, pars triangluaris, and pars orbitalis VOIs. The MNI brain template from FSL was used as a whole-brain VOI. V_T_ also was computed on a voxel-by-voxel basis to create V_T_ maps in MNI space.

V_T_ contains contributions from the number of mGlu5 sites available for specific [^18^F]FPEB binding and includes contributions from nondisplaceable tissue activity and nonspecific binding. The use of a reference region is precluded here as mGlu5 is expressed throughout the human brain (DeLorenzo et al., 2011, Kågedal et al., 2013), including in the cerebellum (Patel et al., 2007).

### Statistical Analyses

Group differences in categorical demographic and substance use variables were tested with Pearson chi-square and Fisher exact tests. Group differences in continuous demographic and substance use variables were assessed with independent *t* tests. Wilcoxon rank-sum tests were used to assess group differences in non-normally distributed variables (age, education, average daily use of methamphetamine, days used in last 30 days, cigarettes smoked per day, days smoked per month).

Group differences in [^18^F]FPEB V_T_ were determined using a general linear model while accounting for individual participant variables, including age, sex, and smoking status. GLM analyses were conducted using [^18^F]FPEB V_T_ in each VOI (whole brain, cortex, thalamus, striatum) as the dependent variable and group (MUD or control), smoking status (smoker or nonsmoker), sex, age, and smoking status and group interaction as independent variables. Main effects were considered significant for α < .05 after Bonferroni correction (α < .05/4). Partial eta squared (η^2^) values were calculated for each model as a measure of effect size (0.01 = small, 0.06 = medium, 0.14 = large). Post-hoc independent *t* tests were used to confirm directionality of main effect findings.

Associations of [^18^F]FPEB V_T_ in the cortex, thalamus, and striatum with measures of methamphetamine use, including average daily use (grams per day), days used per month when actively using, duration of abstinence, and craving on the day of the PET scan, were calculated via partial correlational analyses controlling for age, sex, smoking status, and global proportional scaling (whole brain V_T_). Spearman rank order correlations were used to assess associations with non-normally distributed variables (average daily use, days used per month when actively using, craving). Correlations were considered significant for α < .05 after Bonferroni correction (α < .05/12).

The correlation between [^18^F]FPEB V_T_ in the cortex, thalamus, and striatum and measures of tobacco use, including cigarettes smoked per day, days smoked per month, duration of abstinence, Fagerström score, and Shiffman-Jarvik score, were calculated via partial correlational analyses controlling for age, sex, group, and global proportional scaling. Spearman rank order correlations were used to assess associations with non-normally distributed variables (cigarettes smoked per day, days smoked per month, abstinence duration). Correlations were considered significant for α < .05 after Bonferroni correction (α < .05/15).

Associations of [^18^F]FPEB V_T_ in the dorsolateral prefrontal cortex, inferior frontal gyrus, anterior cingulate cortex, and hippocampus with cognitive performance on the RAVLT, SCAP, and STROOP were calculated via partial correlational analyses, controlling for age, sex, estimated Full-Scale Intelligence Quotient, smoking status, group, and global proportional scaling. Due to the non-normal distribution of SCAP D-prime scores, a Spearman rank order correlation was used to assess its association with V_T_. Correlations were considered significant for α < .05 after Bonferroni correction (α < .05/12).

An exploratory voxel-wise analysis was conducted using SPM12 (https://www.fil.ion.ucl.ac.uk/spm/) to assess the relationship between regional V_T_ and RAVLT performance. Group interactions were not explored due to power limitations. Before analysis, the V_T_ maps were smoothed with a 10-mm FWHM Gaussian filter to reduce noise and improve overlap within individual V_T_ maps. To test for a linear relationship between V_T_ and RAVLT performance independent of group, a multiple regression controlling for sex, age, smoking status, methamphetamine use, and global effects was used. Voxels within any cluster were considered significant at *P* < .001. The cluster formation threshold was set as the expected voxels per cluster (k > 29) designated by SPM or based on probabilistic threshold-free cluster enhancement (pTFCE Toolbox for SPM12; Spisák et al., 2019). Both threshold options are presented.

Differences in MUD and control performance on cognitive tests were assessed via independent *t* tests (RAVLT, STROOP) and Wilcoxon rank-sum tests (SCAP). All statistical analyses were conducted in SAS (Version 9.4).

## RESULTS

Participants in the MUD group reported significantly fewer years of education than participants in the control group (12.7 vs 15.8, t = −4.34, *P *=* *.0002; Table 1). No other significant differences in demographic or substance use variables were found (Table 1; Table 2).

**Figure 1.**
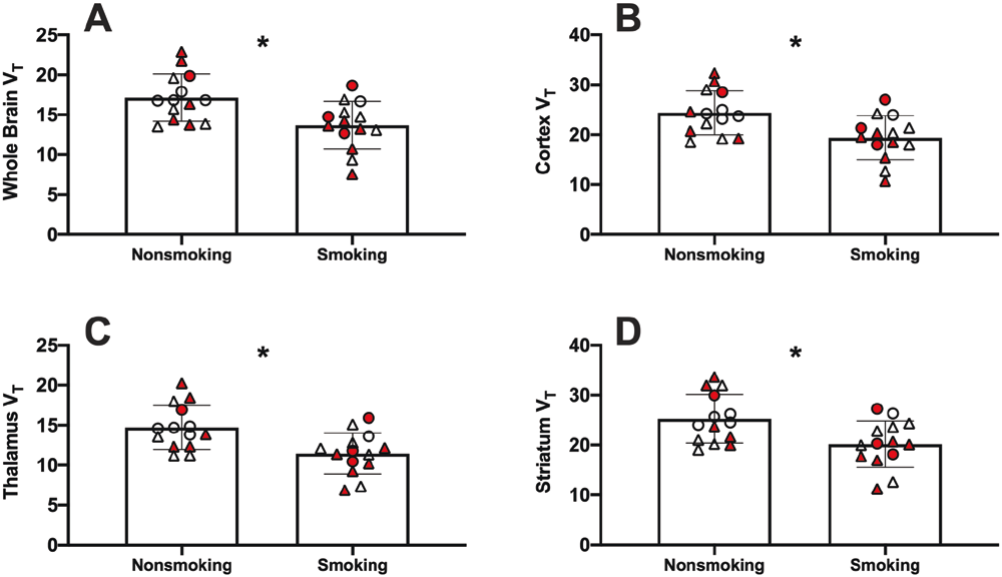
Significant difference in [^18^F]FPEB V_T_ between smoking and nonsmoking participants, independent of MUD. Red-colored symbols represent individuals with MUD, and white represent control participants. Circles represent female participants, and triangles represent male participants.

GLM models examining group differences (MUD vs control), smoking status (smoking vs nonsmoking), sex, age, and the interaction of smoking with group on V_T_ found a significant main effect of smoking on V_T_ in the whole brain (F = 10.25, *P *=* *.0041, semi-partial η^2 ^=^ ^0.22), cortex (F = 10.35, *P *=* *.0040, semi-partial η^2 ^=^ ^0.25), thalamus (F = 11.65, *P = *.0025, semi-partial η^2^ =^ ^0.28), and striatum (F = 8.84, *P *=* *.0070, semi-partial η^2^ = 0.22). No significant effect of MUD group, sex, or age was found. No significant smoking and MUD group interaction was found. V_T_ values in participants who smoked were significantly lower than in nonsmokers in the whole brain (t = 3.08, *P *=* *.0048), cortex (t = 3.0 *P *=* *.0059), thalamus (t = 3.25, *P *=* *.0032), and striatum (t = 2.82, *P* = .0090), independent of MUD group status (Figure 1). V_T_ values for the MUD and control groups, separated by smoking status, are displayed in supplemental Table 1. No significant correlations between measures of methamphetamine or tobacco use and V_T_ were found.

Dorsolateral prefrontal cortical V_T_ showed a significant positive correlation with immediate memory measured by the RAVLT across both groups (r = 0.59, *P *=* *.0039). V_T_ in the inferior frontal gyrus trended toward a significant positive correlation with RAVLT performance as well, but the effect did not survive Bonferroni correction (Table 3). Neither SCAP nor STROOP performance was correlated with V_T_.

**Table 3.** Correlation of [^18^F]FPEB V_T_ in the Prefrontal Cortex With Cognitive Performance

	Dorsolateral prefrontal cortex	Inferior frontal gyrus	Anterior cingulate cortex	Hippocampus
RAVLT Immediate Memory Z-score (r, p)	0.59, 0.0039^*a*^	0.55, 0.0080	0.15, 0.52	−0.14, 0.52
SCAP D-Prime (r_s_, p)	−0.04, 0.88	0.05, 0.83	0.19, 0.45	−0.40, 0.09
Stroop Interference (r, p)	0.16, 0.48	0.19, 0.41	0.12, 0.61	−0.03, 0.90

Abbreviations: RAVLT, Rey Auditory Verbal Learning Test; SCAP, spatial capacity delayed response test.

^*a*^Significant after Bonferroni correction for multiple comparisons and controlling for sex, age, smoking status, participant group, and global effects.

**Figure 2.**
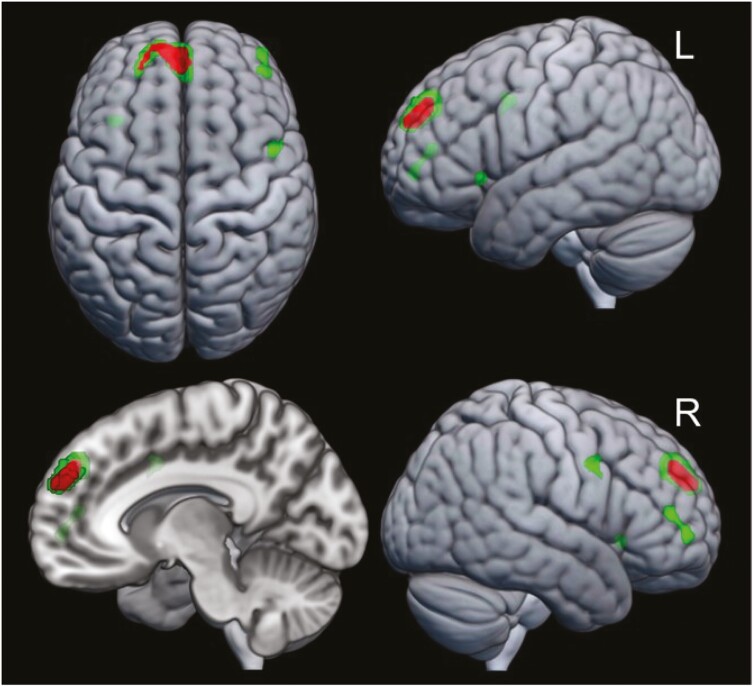
Correlation of [^18^F]FPEB V_T_ with verbal learning. 3D renderings illustrate statistical parametric mapping analyses that tested the linear relationship between [^18^F]FPEB V_T_ and immediate memory on the RAVLT (measured in Z-Scores). Green clusters show voxels demonstrating a significant effect at a threshold of *P* < .001 with a minimum cluster size of k > 29. Significant voxels and clusters, identified using the pTFCE algorithm, are highlighted in red (*P* < .001). Sex, age, smoking status, participant group, and global effects were controlled.

An exploratory voxelwise analysis testing the association between RAVLT and V_T_ identified a significant positive cluster in the dorsolateral prefrontal cortex using the threshold-free cluster enhancement algorithm (Figure 2). Using a minimum cluster size of k > 29, additional positive clusters were found in the superior frontal, rostral middle frontal, caudal middle frontal/precentral gyri, and the insula (Table 4).

**Table 4.** Correlation of RAVLT Performance With [^18^F]FPEB V_T_: Voxelwise Analysis

Location (Desikan-Killiany)	Hemisphere	MNI coordinates peak			Cluster size	Peak level
		X	Y	Z	k Voxels	T Values
Superior frontal^*a*^	Left	−6	54	28	464	6.15
Rostral middle frontal^b^	Right	42	46	12	61	4.35
Caudal middle frontal/ precentral^b^	Right	48	4	38	31	4.27
Insula (anterior)^b^	Left	−32	22	−4	29	4.25

Abbreviations: RAVLT, Rey Auditory Verbal Learning Test; MNI, Montreal Neurological Institute; TFCE threshold-free cluster enhancement.

^*a*^ *P < *.001 TFCE.

*^b^P* < .001 (k = 29).

Performance on the SCAP was significantly worse in the MUD group vs the control group (Z = 2.63, *P *=* *.0065), with lower D-prime values in the MUD group (Table 5). Though not statistically significant, performance on the RAVLT (d = 0.70) and STROOP (d = 0.61) in the MUD group was also below control values. Effect sizes of the group differences were moderate-to-large for all 3 tests. Cognitive performance did not differ as a function of smoking status.

**Table 5.** Cognitive Task Scores (Mean ± SE)

	Control		Methamphetamine use disorder		Group difference (*P*, Cohen d)
	Smoking N = 6	Nonsmoking N = 8	Smoking N = 8	Nonsmoking N = 6	
RAVLT Z-Score	0.65 ± 0.6	0.18 ± 0.3	0.04 ± 0.4	−1.05. ± 0.4	*P *=* *.0756, d = 0.70
SCAP D-Prime^*a*^	3.3 ± 0.5	2.6 ± 0.3	2.0 ± 0.4	1.7 ± 0.2	*P* = .0065, d = 1.03
STROOP interference	8.3 ± 3.6	9.0 ± 4.4	2.6 ± 1.8	5.6 ± 1.6	*P = *.1301, d = 0.61

Abbreviations: MUD, methamphatamine use disorder; RAVLT, Rey Auditory Verbal Learning Test; SCAP, spatial capacity delayed response test.

^*a*^Significant group difference, control vs MUD (*P* < .05).

## DISCUSSION

In this first assessment, to our knowledge, of mGlu5 in human participants with MUD, we found no significant difference in global or regional mGlu5 in individuals with MUD abstinent from methamphetamine for 19–75 days (average = 42 days) compared with control participants. These findings differ from the below-control mGlu5 receptor availability in individuals with moderate-to-severe cocaine dependence who were actively using the drug when tested (Martinez et al., 2014; Milella et al., 2014). Participants who did not smoke cigarettes exhibited globally higher V_T_ for [^18^F]FPEB than those who smoked, extending previous findings of lower mGlu5 availability in individuals who smoke (Akkus et al., 2013, 2017; Hulka et al., 2014; Baldassarri et al., 2023). Across participants (but controlling for group status), short-term verbal memory performance evaluated by the RAVLT correlated positively with V_T_ in the dorsolateral prefrontal cortex and superior frontal gyrus, while no association was found between V_T_ and visual working memory performance or inhibitory control.

Lack of an mGlu5 deficit in participants with MUD may reflect the recovery of mGlu5 during abstinence. In the aforementioned studies of cocaine dependence (Martinez et al., 2014; Milella et al., 2014), the maximum duration of abstinence from cocaine was 14 days and duration of abstinence correlated with mGlu5 availability in the striatum, amygdala, and insula (Milella et al., 2014). In our sample of participants with MUD, the association between duration of methamphetamine abstinence and V_T_ in the striatum showed a nonsignificant trend (r_s_ = 0.56, *P *=* *.073, uncorrected). The possibility of recovery of mGlu5 with abstinence from methamphetamine is consistent with preclinical findings that mGlu5 expression in the nucleus accumbens is decreased following 2 weeks of cocaine self-administration but not at 60 days after the drug is withdrawn (Ben-Shahar et al., 2009).

Our study population was more heterogeneous in drug use and mental health status than participants in prior studies assessing cocaine dependence. Both Martinez et al. and Milella et al. recruited participants who met criteria for cocaine dependence but no other DSM-5 Axis I diagnosis, whereas this study included individuals with polydrug use and histories of depression, anxiety, obsessive compulsive disorder, and bulimia nervosa. More inclusive criteria used here are consistent with capturing changes in drug use behaviors over the past decade, particularly the increase in polydrug use by individuals using stimulants and opioids (Strickland et al., 2021), and consideration of psychiatric co-morbidities. These co-morbidities warrant individual consideration as alcohol use, depression, and anxiety have all been linked to mGlu5 (reviewed in Asch et al., 2023). Notably, in one study (Baldassarri et al., 2023), individuals with co-occurring major depressive disorder and tobacco use did not significantly differ in mGlu5 availability from those with major depressive disorder alone, though individuals with co-occurring PTSD and tobacco use exhibited higher mGlu5 availability in the orbitofrontal, dorsolateral prefrontal, and anterior cingulate cortex than individuals with PTSD alone.

Smoking was associated with lower V_T_ globally in this sample, paralleling the findings of Hulka et al. (2014), in which smoking was associated with lower mGlu5 availability but cocaine use was not. The effect of smoking on mGlu5 availability has been replicated multiple times, with lower receptor availability in individuals who smoked cigarettes (Akkus et al., 2013, 2016, 2017; Hulka et al., 2014; Baldassarri et al., 2023). Smoking has also been associated with lower mGlu5 availability in individuals with cocaine dependence (Martinez et al., 2014) and alcohol use disorder (Hillmer et al., 2021).

Notably, the control group in Milella et al. (2014) contained only 1 current smoker, suggesting that the higher mGlu5 availability in control participants compared with those with cocaine dependence could reflect the lack of participants who smoke. Some studies have also reported associations between mGlu5 availability and smoking-related variables, such as duration of smoking (Akkus et al., 2013) and duration of abstinence (Hulka et al., 2014). However, mGlu5 availability did not correlate with measures of nicotine consumption or severity of nicotine dependence (Akkus et al., 2013; Hulka et al., 2014). We also found no significant associations between the duration of abstinence (even when excluding 1 participant who had abstained for 17 days) or any other smoking-related variable with V_T_. The generally light-to-moderate levels of smoking in our participants could contribute to the lack of association between smoking variables and V_T_. Average Fagerström scores in these participants were under 3, representing low nicotine dependence (Table 1), with several participants reporting less than daily smoking. This suggests that even low levels of tobacco use are associated with low mGlu5 availability, replicating the finding that both light and heavy smokers exhibit low mGlu5 availability compared with never-smokers (Hulka et al., 2014) and a main effect of smoking in a study with individuals smoking fewer than 6 cigarettes per day on average (Martinez et al., 2014).

The positive association between prefrontal cortical V_T_ and performance on the RAVLT observed here extends previous findings that link mGlu5 availability to cognitive performance in humans (Mecca et al., 2020; Régio Brambilla et al., 2020; Esterlis et al., 2022; Wang et al., 2024). Global cognition (Mecca et al., 2020; Wang et al., 2024), episodic memory (Mecca et al., 2020), processing speed (Régio Brambilla et al., 2020), and attention (Esterlis et al., 2022) all have been positively associated with mGlu5 availability measured by PET, though the radiotracers and patient populations have differed among studies. However, working memory, executive function, and verbal learning did not associate with mGlu5 V_T_ in a recent study including participants with PTSD, major depressive disorder, and healthy controls (Esterlis et al., 2022). Despite these conflicting findings, the link between cognition and mGlu5 function is well-supported preclinically, with deletion or knockout of the mGlu5 receptor producing impairment of spatial (Jia et al., 2001) and inhibitory learning (Xu et al., 2009) in rodents.

Pharmacological studies have also linked mGlu5 to cognition. In rodents, MPEP (2-Methyl-6-(phenylethynyl)pyridine), an mGlu5 antagonist, impaired working and spatial memory (Simonyi et al., 2010; Clifton et al., 2013), whereas positive allosteric modulators of mGlu5 have produced improvements in spatial memory (Balschun et al., 2006) and reaction time (Liu et al., 2008; Uslaner et al., 2009). Taken together, these findings suggest that development of pharmacotherapies targeting mGlu5 to ameliorate cognitive impairment, which interferes with behavioral treatment for MUD, may be of particular interest (Petzold et al, 2021).

There are some limitations in this study, of which sample size is one, that may have affected our ability to detect sex-specific effects. Whereas preclinical data have suggested [^11^C]ABP688 binding potential (Smart et al., 2019) and effects of methamphetamine exposure on mGlu5 expression (Denning et al., 2024) may be sex dependent, no effect of sex was observed in this study.

Another limitation is the inclusion of participants with polydrug use, which may have affected the findings. Additionally, the inclusion of only treatment-seeking participants may add a selection bias, since only participants already willing to abstain enrolled in this study. Recruitment methods did not require enrollment in a treatment program, but all potential participants who were not receiving treatment failed to progress past screening procedures. Efforts were made to recruit individuals who smoked cigarettes to the control group to match smoking in the MUD group. The percentages of individuals who smoked in the 2 groups did not significantly differ, but the control group had fewer smokers than the MUD group. Additionally, several “nonsmokers” in the MUD group and 1 nonsmoker in the control group had formerly smoked. Although mGlu5 availability recovers after 6 months of abstinence in former smokers (Akkus et al., 2016), several of the participants had abstained from cigarettes for a shorter period.

The state of smoking abstinence may be a consideration. While all scan procedures were performed in a state of abstinence from nicotine and other substances of abuse to ensure measurement of accurate mGlu5 V_T_, participants were not abstinent from nicotine during cognitive testing. Nicotine has positive effects on cognitive function, particularly memory and attention (reviewed in Rezvani and Levin, 2001; Heishman, Kleykamp and Singleton, 2010), and withdrawal produced deficits (reviewed in Heishman, Kleykamp and Singleton, 2010; Ashare, Falcone and Lerman, 2014). Participants in this study who smoked primarily reported light-to-moderate cigarette use, with average Fagerström scores under 3.3 indicating low dependence (Table 2). However, a small percentage of smokers had higher scores (5–8). The degree of nicotine dependence influences the effect of smoking and withdrawal on cognitive performance (Tait et al., 2000; Nesic et al., 2011). Heavy smokers perform worse on a short-term memory task during withdrawal than light smokers, with smoking ameliorating this difference (Tait et al., 2000). Cognitive testing was performed in a non-abstinent state to avoid any impact of withdrawal on cognitive performance.

## Supplementary Material

pyae031_suppl_Supplementary_Tables

## Data Availability

All self-report, cognitive test, and summary PET data discussed in this manuscript are publicly available from the Open Science Framework web site under project title, “Brain mGlu5 is linked to cognition and cigarette smoking, but does not differ among those abstinent from chronic methamphetamine use and controls.”

## Interest Statement

L.J.M. has received research support from Aelis Farma.
