# Supplementary material for: Brain mGlu5 Is Linked to Cognition and Cigarette Smoking but Does Not Differ From Control in Early Abstinence From Chronic Methamphetamine Use
Source: Int J Neuropsychopharmacol. 2024 Aug 9;27(8):pyae031. doi: 10.1093/ijnp/pyae031 (PMC11348008; doi:10.1093/ijnp/pyae031)
Supplement: pyae031_suppl_Supplementary_Tables [file pyae031_suppl_supplementary_tables.docx]

**Supplemental Table 1. [^18^F]FPEB V_T_ values (Mean ± Standard Error).**

|  | **Control** | | **Methamphetamine Use Disorder** | |
| --- | --- | --- | --- | --- |
|  | *Smoking*  *n = 6* | *Nonsmoking*  *n = 8* | *Smoking*  *n = 8* | *Nonsmoking*  *n = 6* |
| **Whole Brain** | 14.3 ± 3.6 | 16.4 ± 0.7 | 13.2 ± 1.1 | 18.2 ± 1.6 |
| **Cortex** | 20.1 ± 1.8 | 23.1 ± 1.2 | 18.8 ± 1.7 | 26.0 ± 2.2 |
| **Striatum** | 21.6 ± 2.0 | 24.1 ± 1.5 | 19.1 ± 1.6 | 26.8 ± 2.4 |
| **Thalamus** | 12.0 ± 1.1 | 14.0 ± 0.8 | 11.0 ± 0.9 | 15.7 ± 1.4 |
| **Dorsolateral Prefrontal Cortex** | 20.3 ± 1.7 | 23.4 ± 1.3 | 19.1 ± 1.7 | 26.2 ± 2.3 |
| **Inferior Frontal Gyrus** | 20.8 ± 1.8 | 24.1 ± 1.3 | 19.7 ± 1.7 | 27.0 ± 2.2 |
| **Anterior Cingulate Cortex** | 23.1 ± 2.1 | 27.8 ± 1.5 | 22.5 ± 2.1 | 30.3 ± 2.6 |
| **Cerebellar White Matter** | 3.8 ± 0.4 | 4.3 ± 0.3 | 3.3 ± 0.3 | 4.7 ± 0.4 |

**Supplemental Table 2. [^18^F]FPEB dose characteristics (Mean ± Standard Error).**

|  | **Control** | | **Methamphetamine Use Disorder** | |
| --- | --- | --- | --- | --- |
|  | *Smoking*  *n = 6* | *Nonsmoking*  *n = 8* | *Smoking*  *n = 8* | *Nonsmoking*  *n = 6* |
| **Injected dose (mCi)** | 4.89 ± 0.08 | 5.41 ± 0.26 | 4.95 ± 0.09 | 5.06 ± 0.08 |
| **Specific activity at time of injection (Ci/µmol)** | 12.2 ± 1.7 | 14.1 ± 2.3 | 14.7 ± 3.5 | 13.1 ± 3.3 |
| **Mass dose (µmol)** | 0.00046 ± 0.0001 | 0.00045 ± 0.0001 | 0.00066 ± 0.0003 | 0.00051 ± 0.0001 |
| **Injected dose (mCi/kg)** | 0.056 ± 0.006 | 0.066 ± 0.004 | 0.064 ± 0.004 | 0.063 ± 0.004 |
